# MreB: unraveling the molecular mechanisms of bacterial shape, division, and environmental adaptation

**DOI:** 10.1186/s12964-025-02373-y

**Published:** 2025-08-22

**Authors:** Yaqi Wang, Yalan jiang, Zhixuan Song, Chengbin Zhu, Yujun Tang, Jiaofeng Peng, Peng Liu

**Affiliations:** 1 Institute of Pathogenic Biology, Basic Medical School, Hengyang Medical School, University of South China, Hengyang, 421001 China; 2 Affiliated Hengyang Hospital of Hunan Normal University &Hengyang Central Hospital, Hengyang, 421001 Hunan China; 3 Plastic Surgery Department, The Second Hospital University of South China, Hengyang, 421001 China; 4 Hengyang Chinese Medicine Hospital, Hengyang, 421001 Hunan China

**Keywords:** MreB, Bacterial cytoskeleton, Cell wall synthesis, Morphogenesis, Cell polarity

## Abstract

As a key bacterial actin-like protein, MreB plays crucial roles in maintaining cell shape, regulating peptidoglycan synthesis, and coordinating chromosome segregation, making it a promising target for novel antibiotics. This review comprehensively explores MreB’s molecular architecture, its assembly into antiparallel protofilaments, and its pivotal roles in bacterial cell morphology and division. We also delve into how MreB interacts with membrane-associated proteins such as RodZ and MreC/D to coordinate cell wall synthesis and respond to environmental signals like ion gradients and temperature changes. Furthermore, we highlight the cooperation and functional divergence between MreB and FtsZ, underscoring the evolutionary adaptability of bacterial cytoskeletal structures. The structural and functional parallels between MreB and eukaryotic cytoskeletal proteins are also examined, offering new insights into the evolution of cytoskeletal systems. By integrating insights from structural biology, synthetic biology, and microbial ecology, this review aims to provide a deeper understanding of MreB’s role in bacterial biology, its dynamic responses to environmental cues, and its implications for therapeutic innovation. This comprehensive analysis not only enhances our knowledge of bacterial self-organization mechanisms but also paves the way for the development of innovative antimicrobial strategies to address the growing challenge of antibiotic resistance.

## Introduction

The cytoskeleton, a dynamic protein network orchestrating cellular organization and mechanics, has long been considered a hallmark of eukaryotic systems. Among the components, actin is distinguished by its function in activities like motility and cytokinesis, facilitated by ATP-driven polymerization into polarized filaments called F-actin [1, 2]. However, the discovery of MreB in 2001 revealed that bacteria also possess organized structural frameworks [3, 4]. As a bacterial actin homolog, MreB shares structural homology with eukaryotic actin, including conserved ATP-binding motifs and protofilament assembly, while it has evolved distinct functional adaptations. The sequence and structural similarity between MreB and eukaryotic actin were first predicted based on bioinformatics analysis [5]. Unlike actin’s polar filaments, MreB forms antiparallel double-stranded polymers that generate mechanical forces on bacterial membranes, enabling functions such as cell shape maintenance, peptidoglycan synthase positioning, and chromosome segregation [6, 7]. The functional diversification of MreB in bacterial taxa underscores its evolutionary plasticity. In *Escherichia coli* (*E. coli*), MreB depletion causes cells to lose their rod shape and form spheres with division defects [8]. In contrast, *Bacillus cereus* (*B. cereus*) has three MreB paralogs (MreB, Mbl (Metallo-β-lactamase-like), and MreBH) that share functions in cell wall synthesis. Deletion of all three leads to cell rounding and lysis [9]. *Spiroplasma* species utilize multiple MreB homologs, like MreB5, to drive helical movement by creating twists, independent of cell wall synthesis [10, 11]. Pathogens like *Chlamydia* further repurpose MreB to coordinate division in the absence of FtsZ, highlighting its adaptability [11].

FtsZ, a tubulin homolog, orchestrates bacterial division by polymerizing into dynamic filaments analogous to microtubules, while MreB, as an actin-like protein, maintains cell shape. Despite two decades of research, critical gaps remain in our understanding of how MreB integrates mechanical, metabolic, and environmental signals to regulate bacterial physiology. For instance, the mechanism by which MreB filamentous structures sense membrane curvature or dynamically respond to stress remains unresolved. This review synthesizes the current knowledge of the molecular architecture, functional networks, and environmental adaptability of MreB. It aims to elucidate the role of MreB as a multifunctional scaffold in bacterial biology, explore its potential as a target for novel antimicrobial strategies, and identify key unanswered questions that merit future investigation.

## Molecular structure and dynamic assembly

### Phylogenetic conservation and divergence of MreB

To summarize the evolutionary dynamics of the MreB protein family, we constructed a phylogenetic tree covering diverse bacterial taxa, including *Enterobacteriaceae*, *Bacillaceae*, *Spiroplasmataceae*, and key pathogens like *Mycobacterium* and *Pseudomonas*. Our analysis reveals a “conserved core – divergent periphery” pattern. The core regions, including ATP-binding motifs and polymerization interfaces, are highly conserved to maintain essential cytoskeletal functions such as cell shape. In contrast, significant diversification occurs through C-terminal modifications, gene duplication, and horizontal gene transfer.

**Fig. 1:**
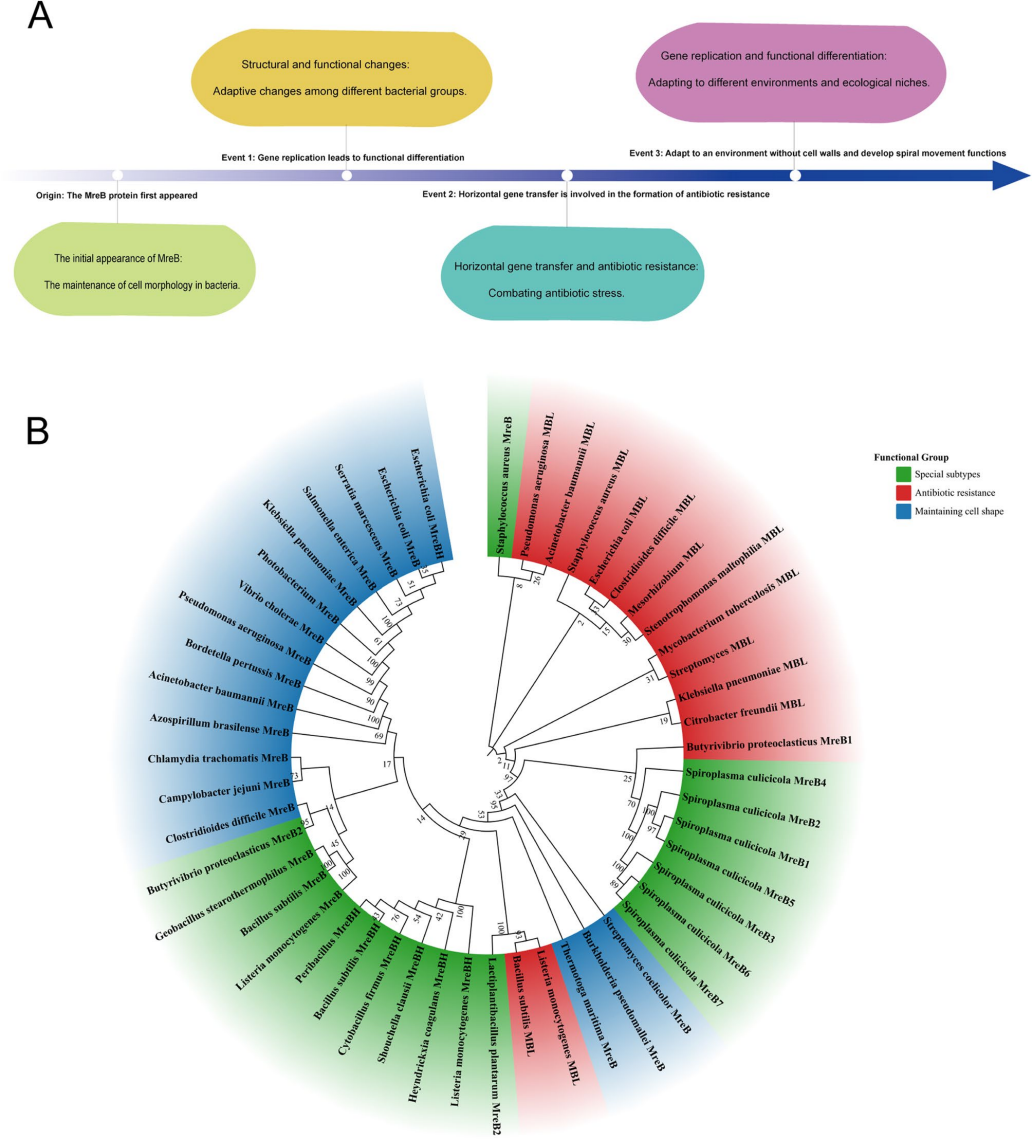
Discovery History and Evolutionary Tree of MreB Protein Functions.** A** The timeline, progressing from left to right, begins with the emergence of the MreB protein. Subsequent gene duplication events drove the diversification of MreB’s functions. Horizontal gene transfer then enabled MreB to contribute to the development of antibiotic resistance. Ultimately, MreB adapted to cell wall-less environments and evolved the capacity for helical movement. **B** Maximum-likelihood tree of MreB and MreB-like protein sequences from diverse bacterial species generated using MEGA 11. Branches are color-coded to highlight major functional categories: blue signifies proteins involved in maintaining cell shape; red identifies proteins associated with antibiotic resistance; and green denotes specialized cytoskeletal variants. Bootstrap values ≥ 60% are labeled for major nodes

In the evolutionary history of bacteria, the MreB protein has played a crucial role, with its functional and structural evolution closely tied to bacterial adaptation to various environments. Initially, MreB was identified as a protein responsible for maintaining cell shape in bacteria [12]. Over time, MreB genes underwent duplication events, leading to functional diversification. For example, in some bacteria, MreB proteins became involved in cell wall synthesis and chromosome segregation [4, 13]. Horizontal gene transfer further contributed to the acquisition of new functions, such as antibiotic resistance [11]. In cell wall-less bacteria like spiroplasmas, MreB evolved to drive helical cell movement, facilitating effective locomotion in their specific environments [10, 14] (Fig. 1A).

Three major clades were identified. The first includes classic MreB homologs in Gram-negative rods like *E. coli*, with high sequence conservation (bootstrap ≥ 94). The second is exemplified by *Spiroplasma culicicola*, which has seven MreB paralogs (MreB1–MreB7) forming a robust cluster (bootstrap 100). These paralogs show pronounced functional divergence through subtype specialization: MreB3 lacks the catalytic glutamate and threonine residues required for ATP hydrolysis, likely relegating it to auxiliary functions, whereas MreB5 possesses an extended C-terminal domain enabling lateral interactions. These adaptations collectively facilitate helical cell shape maintenance through antiparallel filament bundling in wall-less bacteria [10, 15–17]. The third clade comprises Metallo-β-lactamase-like (Mbl) proteins, distinct from canonical MreB (bootstrap 97), with minimal sequence homology but strong evidence of horizontal gene transfer across diverse genera, including *Klebsiella pneumoniae*, *Mycobacterium tuberculosis*, and *Staphylococcus aureus* (*S. aureus*), indicating dissemination of antibiotic resistance (Fig. 1B).

Lineage-specific adaptations are also observed, such as the co-clustering of *Bacillus subtilis* (*B. subtilis*) MreBH and *Listeria monocytogenes* Mbl (bootstrap 89) and the independent branching of *S. aureus* MreB, suggesting unique pathogenic adaptations. Overall, MreB’s plasticity enables bacteria to adapt to diverse environments, from maintaining cell shape to driving motility and conferring drug resistance.

### Molecular architecture of MreB: secondary and tertiary structure features

**Fig. 2:**
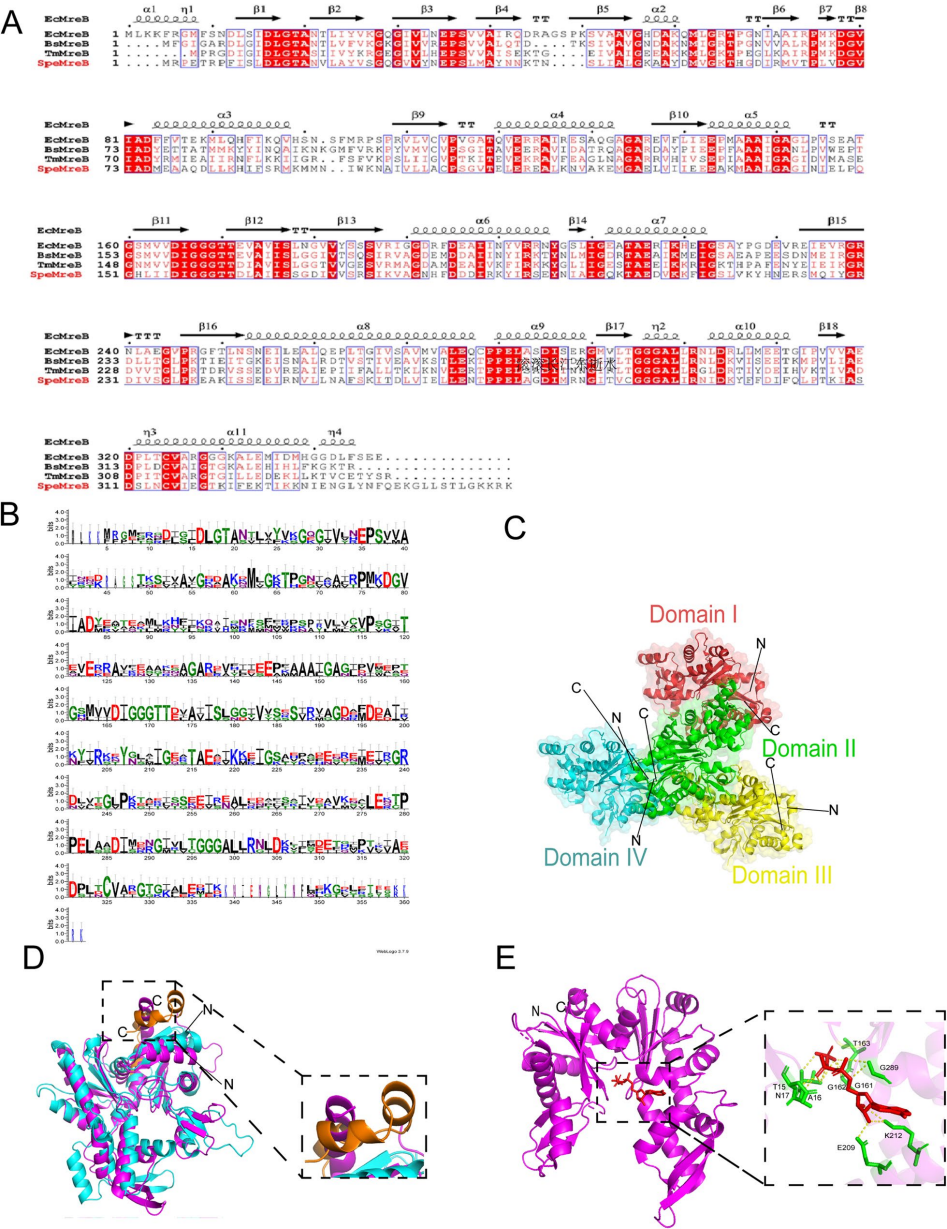
Structural features of MreB and actin. **A** Predicted secondary structures of MreB orthologs from four key bacterial groups: *B. subtilis* (Gram-positive), *E. coli* (Gram-negative), *T. maritima* (extremophile) and *S. citri* (wall-less), as generated by ESPrint 3. 0. **B** Sequence conservation analysis of MreB from the four bacterial groups listed was performed using WebLogo, highlighting the positions of highly conserved amino acid residues. **C** The domain architecture of *B. taurus* actin, which includes domains I through IV, is presented for structural comparison with other proteins. **D** An interaction model between *B. taurus* actin (blue) and *G. stearothermophilus* MreB (purple) was generated using AlphaFold3. Regions of significant structural misalignment are highlighted in orange. **E** The ATP-binding pocket in *G. stearothermophilus* MreB is visualized using PyMOL, with key residues (T15, A16, N17, G161, G162, T163, E209, K212, G289) labeled

Computational analyses across diverse bacterial lineages delineate conserved and divergent structural features of MreB, providing insights into its functional underpinnings. Secondary structure predictions for MreB orthologs from *B. subtilis* (Gram-positive), *E. coli* (Gram-negative), *Thermotoga maritima* (*T. maritima*, extremophile), and *Spiroplasma citri* (*S. citri*, wall-less) using ESPrint 3.0 reveal consistent core elements, including α-helices, β-strands, and notably, conserved N-terminal DLGTA/GXGXG motifs characteristic of ATP-binding domains in actin-like proteins (Fig. 2A). This conservation strongly suggests a fundamental role for nucleotide-dependent regulation in MreB function across evolutionarily distant bacteria.

WebLogo analysis of MreB sequences from these four groups further identifies positions of high amino acid conservation (Fig. 2B). The pronounced conservation of glycine and threonine residues within the N-terminal region aligns precisely with established ATP binding and hydrolysis sites in related proteins. Central hydrophobic patches likely represent interfaces for monomer interaction and polymerization, while conserved C-terminal motifs potentially facilitate the recruitment of partner proteins, such as cell wall synthesis machinery. Importantly, these functional interpretations are based on sequence motif conservation and analogy to actin; definitive roles await experimental confirmation.

For comparative context, the canonical four-domain architecture of eukaryotic actin (*Bos taurus*,* B. taurus*) serves as a structural reference point (Fig. 2C). Computational modeling via AlphaFold3 predicts significant local conformational differences at the interaction interface between *B. taurus* actin and *Geobacillus stearothermophilus* (*G. stearothermophilus*) MreB, highlighted by misaligned regions (Fig. 2D). These structural distinctions plausibly underlie the specialized functions of MreB, such as its propensity to form antiparallel polymers generating circumferential forces for morphogenesis, contrasting with actin’s polarized filaments driving motility.

Critical examination of the *G. stearothermophilus* MreB structure identifies key residues (T15, A16, N17, G161, G162, T163, E209, K212, G289) spatially arranged to form the ATP-binding pocket (Fig. 2E). The location and composition of this pocket, corroborated by evidence from the literature, robustly support its essential function in nucleotide-driven polymerization dynamics, a core mechanism underpinning MreB’s role in bacterial cellular organization [18].

Collectively, these analyses characterize the structural landscape of MreB. The conserved ATP-binding motifs and pocket architecture (Fig. 2A, B, E), combined with predicted polymerization interfaces (Fig. 2B), align with and reinforce the established model of MreB utilizing ATP-dependent filament assembly to orchestrate essential processes like cell shape maintenance. The observed structural divergence from actin (Fig. 2D) correlates well with MreB’s unique functional adaptations, particularly its capacity for antiparallel filament formation and circumferential force generation within the bacterial cell envelope.

### Structural and functional parallels with actin

#### Evolutionary conservation and divergence

MreB and actin exhibit striking structural similarities, including conserved ATP-binding motifs and polymerization dynamics [3]. Functionally, however, their architectures drive distinct mechanical outputs. MreB generates circumferential forces along bacterial membranes to direct cell wall synthesis and morphogenesis, whereas actin’s stably polarized filaments enable eukaryotic motility, vesicle trafficking, and cytokinesis [2, 6]. Notably, MreB’s role in maintaining membrane tension and rigidity exhibits functional convergence with cortical actin’s regulation of lipid raft dynamics in eukaryotes [19]. Further parallels exist in unexplored interactions: MreB coordinates chromosome segregation through RNA polymerase (RNAP) coupling, mirroring nuclear actin’s transcriptional regulatory roles [13].

#### Functional specialization across domains

While both proteins polymerize in an ATP-dependent manner, their roles diverge sharply. In walled bacteria, MreB directs peptidoglycan synthase localization and cell wall synthesis, whereas actin orchestrates cytoskeletal remodeling in eukaryotes. Notably, MreB typically forms antiparallel protofilaments without intrinsic unidirectional polarity unlike eukaryotic actin, recent evidence suggests that under specific conditions, such as at membrane concavities, MreB can exhibit treadmilling behavior with directional polarity [20]. In the absence of stable polarity, MreB compensates through interactions with membrane proteins like RodZ. RodZ anchors peptidoglycan (PG) synthases by forming a bridge complex, for example, with MreC/D, thereby linking the cytoskeleton to cell wall synthesis machinery [7, 21–23]. This functional adaptation exhibits convergence with cortical actin: both regulate membrane rigidity through lipid domain organization [24]. Furthermore, MreB’s coordination of chromosome segregation via RNAP coupling parallels nuclear actin’s involvement in transcriptional regulation, suggesting conserved mechanotransduction pathways across domains [13].

#### Structural features

**Fig. 3:**
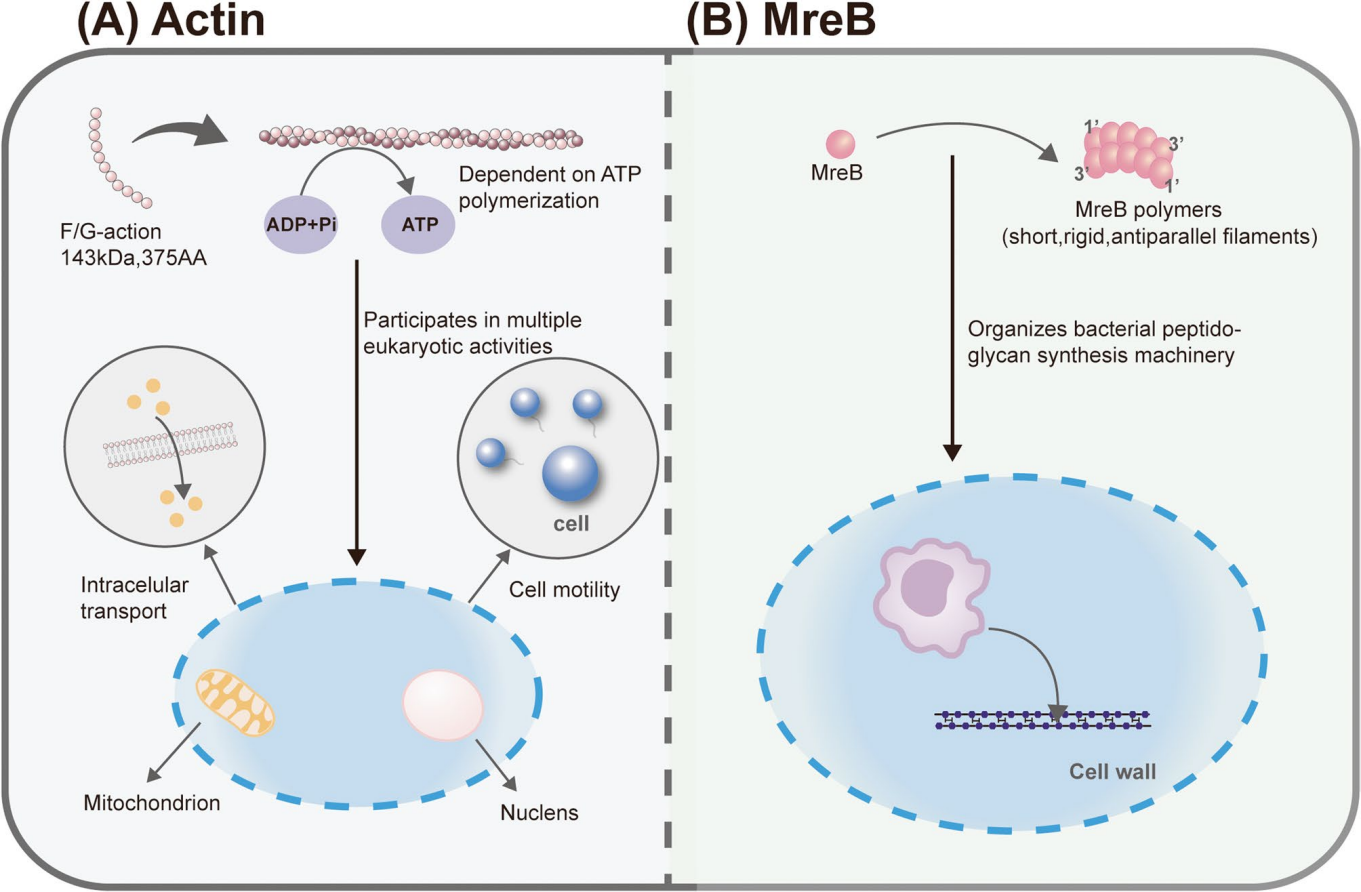
Structural and functional comparison between MreB and eukaryotic actin.** A** Eukaryotic Actin System: G-actin monomers polymerize ATP-dependently into polarized double-helical F-actin filaments, undergoing dynamic cycles regulated by ATP hydrolysis. Actin drives cell motility, cytokinesis, vesicle trafficking, and intracellular transport. **B** Bacterial MreB System: MreB monomers assemble via ATP binding into short, rigid, single-stranded helical filaments. MreB maintains bacterial shape by coordinating peptidoglycan synthesis enzymes during cell wall elongation

The eukaryotic actin monomers (G-actin) are 43 kDa globular proteins composed of 375 amino acids, which polymerize via ATP-dependent processes into polarized, double-helical filaments (F-actin), with dynamic assembly regulated by ATP hydrolysis [25–27]. Although both MreB and actin exhibit ATP-binding capacity and dynamic polymerization, key differences distinguish them. Structurally, MreB forms shorter, rigid anti-parallel filaments, whereas actin microfilaments adopt unidirectional helical polarity [4, 6, 28]. Functionally, MreB specializes in spatially organizing bacterial peptidoglycan synthesis machinery, while actin participates in diverse eukaryotic processes such as cell motility, intracellular transport, and cytoskeletal remodeling [2, 29] (Fig. 3). These divergences in structural dimensions and functional specialization underscore the evolutionary divergence between the cytoskeletal systems of prokaryotes and eukaryotes.

### Functional redundancy and specialization of MreB paralogs

**Fig. 4:**
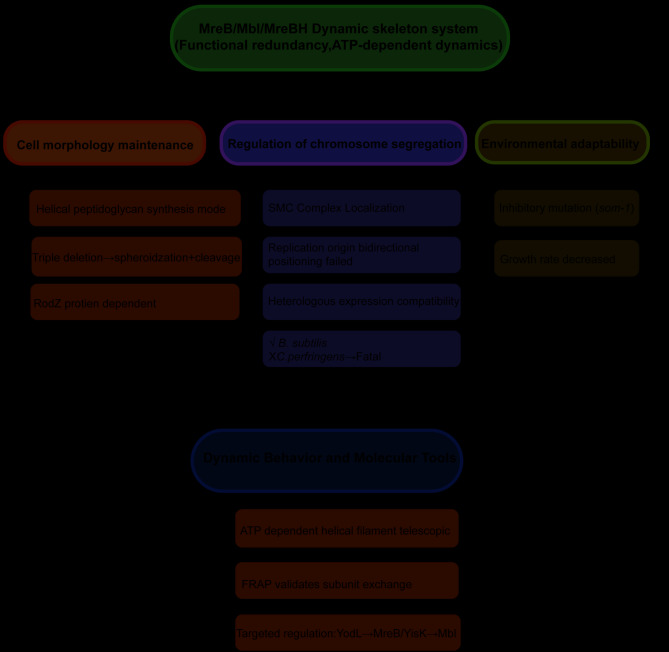
Integrated description of the MreB/Mbl/MreBH cytoskeletal system. The MreB/Mbl/MreBH cytoskeletal system orchestrates critical bacterial functions through functional redundancy and ATP-dependent dynamic behaviors, enabling multidimensional regulation. Central to cell morphogenesis, this system coordinates helical peptidoglycan synthesis and interacts with RodZ; its triple deletion (MreB/Mbl/MreBH) disrupts cell shape maintenance, leading to spherical morphology and lysis. Concurrently, it regulates chromosome segregation by positioning the SMC complex, ensuring bidirectional origin localization during replication, and exhibiting compatibility with heterologous expression systems. Environmental adaptability is mediated via stress-responsive mechanisms, including suppressor mutations such as *som-1*, and growth rate modulation under adverse conditions. Dynamic ATP-driven filament extension/contraction underpins its mechanical plasticity, validated by FRAP-based subunit exchange assays. Targeted regulation is achieved through dedicated pathways, such as YodL regulating MreB and YisK regulating Mbl, highlighting its integration into broader cellular networks

In *B. subtilis*, MreB and its homologous proteins Mbl and MreBH constitute a dynamic cytoskeletal system, which is crucial for maintaining cell morphology and chromosome segregation. Studies have revealed that the protein encoded by the Mbl gene exhibits 53% sequence similarity with *E. coli* MreB (EcMreB) and shares 86% sequence with *B. cereus* MreB, suggesting that its function is conserved [30]. Although the inactivation of Mbl does not directly affect cell viability or sporulation, it leads to a reduced growth rate, morphological distortion, and the emergence of intergenic suppressor mutations (such as *som-1*) to compensate for the defects. Further studies indicate that MreB and Mbl regulate chromosome segregation by forming dynamic submembrane helical filaments. Their absence disrupts the subcellular localization of SMC (structural maintenance of chromosomes) complexes, leading to the failure of bipolar localization at the replication origin and even unidirectional movement [30, 31]. In *B. subtilis*, the proteins MreB, Mbl, and MreBH all help control the helical pattern of peptidoglycan synthesis. Even if one of them is mutated, the cell can still maintain rod shape. But if MreB is depleted in an Mbl mutant, or if all three proteins are depleted, the cell wall’s peptidoglycan synthesis is disrupted, causing the cell to become spherical and lyse. This redundancy extends to heterologous expression systems. MreB from *Bacillus licheniformis* can partially substitute for its homolog in *B. subtilis*. Conversely, MreB from *Clostridium perfringens* can cause cell death in *B. subtilis* by disrupting the endogenous cytoskeleton [32]. Dynamic behavior studies have shown that these three proteins form ATP-dependent spiral filaments capable of extension and contraction within seconds; their subunit turnover properties have been confirmed by Fluorescence Recovery after Photobleaching (FRAP) [33]. Specifically, MreB can induce membrane protrusions in *Drosophila* Schneider 2 cells, suggesting that it possesses mechanical functions similar to those of eukaryotic actin [34]. Additionally, the localization of membrane proteins such as RodZ depends on the scaffold effect of MreB/Mbl, confirming that this cytoskeletal system has the ability to recruit proteins [34]. Functional specificity studies have revealed that YodL and YisK can target MreB and Mbl, providing tools to reveal their molecular mechanisms [35]. These findings collectively reveal that *B. subtilis* constructs a robust regulatory network through the dynamic coordination of multiple actin-like proteins, which plays a critical role in maintaining morphogenesis and chromosome organization (Fig. 4).

### The polymerization characteristics of MreB

MreB can polymerize to form filamentous structures, which are essential for maintaining cell shape, synthesizing the cell wall, organizing chromosomes and establishing cell polarity [36]. Although *S. citri* MreB5 (ScMreB5) can form filaments in different nucleotide states, efficient polymerization and filament organization require the ATP-bound state. Conversely, ATP hydrolysis (metabolism) regulates filament dynamics and triggers disassembly. The impaired hydrolysis in the E134A mutant, which locks the protein in the ATP-state, dramatically reduces polymerization efficiency, demonstrating this dual dependence [27]. Studies on *B. subtilis* MreB (BsMreB) further revealed that in vitro polymerization requires millimolar concentrations of divalent cations such as Mg²⁺ and Ca²⁺, and acidic pH value, while being inhibited by low temperatures or high concentrations of monovalent ions such as K⁺ and Na⁺. Intriguingly, although BsMreB exhibits ATP/GTP binding and hydrolytic activity, its critical concentration for polymerization (~ 900 nM) remains unaffected by the type of nucleotide or binding state [37]. This contrasts sharply with *Marine thermophile (Thermotoga maritima)* MreB1, which strictly requires ATP for polymerization and is highly temperature-sensitive [38]. Such interspecies divergence suggests that MreB dynamics in vivo likely depend on cofactors such as cytoskeletal regulators and microenvironmental cues such as membrane potential and metabolite gradients, which may fine-tune its activity [37].

It is noteworthy that the antiparallel polymerization mode of MreB protofilaments is a key feature distinguishing it from eukaryotic actin. Crystallographic studies have shown that the *Caulobacter* MreB protofilament dimer assembles in an antiparallel arrangement and maintains cell shape through tight binding to the membrane. The antibiotics A22 and MP265 inhibit MreB function by binding to regions adjacent to the nucleotide-binding site of MreB, preventing ATP hydrolysis and destabilizing the protofilament dimer [39]. Critical studies reveal that A22 exhibits MreB-independent cytotoxicity and growth inhibition influenced by drug concentration, medium conditions, and bacterial species. In contrast, the structural analog MP265 maintains equivalent MreB disruption efficacy while significantly reducing off-target toxicity. This enhanced specificity allows MP265 to serve as a superior probe for reversible MreB inhibition studies, as demonstrated in *Caulobacter crescentus* (*C. crescentus*) models where its withdrawal restores rod morphology via peptidoglycan remodeling [40].

### Membrane-Binding properties of MreB

The MreB protein has the ability of membrane-binding, and this ability is crucial for its biological functions. The analysis of MreB’s membrane binding characteristics shows that its nucleotide state regulates membrane interactions, and the key catalytic sites also regulate membrane interactions. Studies have shown that the membrane-binding ability of ScMreB5 is regulated by the ATP binding state and allosterically regulated by the ATP hydrolysis state. ScMreB5^WT^ can form filamentous structures under nucleotide-independent conditions. The ATPase-deficient mutant ScMreB5^E134A^ can also form filaments, but the mutant shows significant defects in lateral interaction, which leads to the disorder of filamentous structure organization. Catalytic glutamate residues (Glu134) have been proven to have dual functions. On the one hand, it senses the ATP-bound filament assembly state in this way; on the other hand, it may trigger filamentous depolymerization by promoting ATP hydrolysis. It is worth noting that the mutation at the Glu134 site leads to differential changes in the liposome binding ability, and the type of nucleotide bound can also cause such changes. This indicates that this site regulates the interaction between MreB and the membrane through allosteric mechanisms. These findings suggest that MreB retains the conserved ATP-dependent polymerization mechanism shared with the actin family. However, it was reprogrammed to dynamically regulate the assembly of the filamentous network on the membrane surface through nucleotide state transitions. It also regulates the organization of the filament network on the membrane surface [41].

## Effect of MreB on cell structure and function

### Cell wall and morphological maintenance

#### MreB coordinates peptidoglycan synthesis and cell shape determination

MreB is the central orchestrator of bacterial morphogenesis by spatially organizing the PG synthesis machinery, establishing the structural framework governing cell shape. In rod-shaped bacteria such as *E. coli* and *B. subtilis*, MreB assembles into short, membrane-associated filaments that adopt circumferential orientations perpendicular to the long cell axis [22, 31]. Super-resolution microscopy reveals that these filaments undergo rotational dynamics along the inner membrane, with their migration speed inversely correlating to cell width (∼85 nm/s in wild-type vs. ∼45 nm/s in ΔRodZ mutants) [32, 38, 39]. This mechanical feedback regulates diameter homeostasis [42]. At the molecular level, MreB anchors PG synthesis enzymes (such as penicillin-binding protein 2, PBP2) through direct interaction with RodZ, forming discrete PG assembly centers that coordinate lateral wall synthesis [7, 8, 22]. Notably, this system exhibits significant evolutionary plasticity. While *C. crescentus* MreB drives de novo rod-shape establishment, MreB depletion in spherical bacteria like *S. aureus* disrupts cell wall synthesis and enlarges cell size but does not alter spherical morphology, reflecting its role in cell expansion rather than shape determination.

While some interesting phenomena are presented in the *Mollicutes* class, which encompasses four phylogenetic categories—*Spiroplasma*, *Hominis*, *Pneumoniae*, and *Acholeplasma*/*Anaeroplasma*/*Phytoplasma* [43, 44]. These organisms impact the health of both animals [45–50] and plants [51]. For instance, *Mycoplasma pneumoniae* is a prevalent atypical pneumonia pathogen [52–55]. Additionally, *Mycoplasm hominis* [56], *Mycoplasm genitalium* [57, 58], and *Ureaplasma urealyticum* [59] cause urogenital infections and are associated with various pregnancy complications [60]. Having evolved from Gram-positive bacterial ancestors, *Mycoplasma* have dispensed with their peptidoglycan layer and undergone genome reduction, resulting in a distinctive wall-less structure [61, 62]. Meantime, *Mycoplasma* exhibit gliding motility despite the absence of MreB in their genome. *Spiroplasma*, a member of *Mollicutes*, characterized by the absence of a cell wall and the presence of a mere membrane bilayer delineating its internal and external environments, represents one of the simplest and smallest prokaryotic organisms identified to date. *Spiroplasma* relies on MreB5 to transform from a rod-shaped form to a spiral form. The depletion of MreB5 results in the loss of helicity and motility, while its complementation restores kink propagation and cell elongation [10, 40, 63]. This system operates through MreB5-fibril interactions, in which antiparallel protofilaments generate mechanical forces to alter cellular chirality—a mechanism that underscores the species-specific regulatory network [10, 64, 65]. They are prime examples of the minimalistic adaptation of cytoskeletal systems in wall-less environments [44, 61]. The heterologous expression of *Spiroplasma* MreB5 in *Mycoplasm capricolum* bestows a helical morphology and kink propagation, emulating *Spiroplasma*’s motility-related deformations [66]. However, these recombinant cells failed to achieve directional swimming in liquid media, even when co-expressed with fibril [66]. Cryo-electron microscopy confirmed MreB5 filaments anchored to the plasma membrane through direct lipid interactions [17, 65]. However, the lack of coordinated movement implies that extra *Spiroplasma*-specific elements, such as polar-anchored structures or regulatory proteins, are needed to change local membrane deformations into translational movement [66]. This modular reconstitution underscores MreB5’s capacity to independently apply helicity and membrane dynamics, while emphasizing the necessity of complementary factors for full motility, reflecting the evolutionary adaptations of the cytoskeletal systems in the bacterial lineages. Furthermore, the physical coupling between MreB and the division protein FtsZ achieves spatiotemporal decoupling of the prolonging and division phases, providing a dual guarantee for the fidelity of morphogenesis [67].

#### Multilayered regulatory networks governing cell wall homeostasis

The MreB system integrates mechanochemical signals with metabolic regulation to maintain the dynamic balance of the cell wall. In *B. subtilis*, the three MreB paralogs (MreB, Mbl, and MreBH) exhibit functional redundancy in PG synthesis. However, their combined deletion disrupts lateral cell wall assembly, revealing their cooperative roles in environmental adaptation [9]. The biophysical properties of the membrane regulate MreB dynamics through multiple mechanisms. Flotillin-mediated lipid raft formation enhances the fluidity of MreB filaments, while anionic phospholipids restrict their spatial distribution via electrostatic repulsion [68, 69]. Single-molecule tracking reveals distinct kinetic behaviors: MreB exhibits sustained rotational movement guiding cell wall synthesis, whereas PG enzymes transiently bind to insertion sites for glycan chain incorporation [70]. This dynamic coupling operates through mechanochemical feedback: the MreB-RodZ complex senses membrane curvature changes induced by PG synthesis and adaptively adjusts filament trajectories [7, 71]. Post-translational modifications add regulatory complexity: acetylation reduces the size of the PG synthesis zone to fine-tune cell diameter, while phosphorylation may reprogram MreB activity under stress conditions [72].

#### MreB-Driven morphological diversification in bacterial evolution

The modular architecture of the MreB system enables remarkable morphological diversification across bacteria. In *Streptomyces coelicolor* MreB, originally utilized for vegetative hyphae, was repurposed during the developmental transition period to mediate spore-specific cell wall remodeling, demonstrating functional plasticity during morphogenesis [73]. *C. crescentus* exemplifies localized mechanical adaptation by redistributing divisome components to stalk synthesis regions [74]. Pathogenic *Chlamydia* represents an extreme case: despite lacking canonical FtsZ, it has evolved an alternative division mechanism via MreB-lipid II synthase interactions to adapt to intracellular parasitic life [11]. Similarly, *Spiroplasma* employs MreB isoforms (MreB1-5) to coordinate the shape and movement of helical cells. Heterologous expression of MreB4-MreB5 or MreB1-MreB5 in the minimal synthetic bacteria (JCVI-syn3B) recapitulated the helical morphology and swimming, demonstrating MreB’s role as a minimal motility module [75]. Additionally, MreB5 interacted with host Rab7 GTPase during *Spiroplasma eriocheiris* (*S. eriocheiris*) infection, enhancing phagosome fusion and limiting intracellular replication, revealing its dual roles in pathogenicity and cytoskeletal regulation [76].

The functional plasticity of MreB is further exemplified in *S. eriocheiris*, a wall-less bacterium. Studies have shown that MreB5 isoforms (e.g., MreB4-MreB5 and MreB1-MreB5) are essential for maintaining helical morphology and generating kink propagation, which drives motility in viscous environments [77]. This modular reconstitution underscores MreB5’s capacity to independently apply helicity and membrane dynamics, while emphasizing the necessity of complementary factors for full motility, reflecting the evolutionary adaptations of the cytoskeletal systems in the bacterial lineages. Intriguingly, MreB loss creates evolutionary trade-offs. Its absence hinders growth efficiency and disrupts β-lactam antibiotic targeting by compromising cell wall integrity, providing new insights into the evolution of antibiotic resistance [78].

### Cell membranes and dynamic regulation

#### Membrane anchoring of MreB: structural insights and species-specific adaptations

The MreB cytoskeleton orchestrates bacterial morphogenesis through dynamic membrane interactions. Structural studies reveal species-specific membrane-binding strategies: *T. maritima* MreB (TmMreB) employs membrane-insertion loops for anchoring, whereas EcMreB utilizes an N-terminal amphiphilic helix [6]. Despite mechanistic divergence, functional conservation is evident.

In *B. subtilis*, the heterologously expressed MreB autonomously assembles into membrane-associated filaments [34]. Super-resolution microscopy demonstrates that these filaments adopt dynamic helical geometries, measuring ~ 3.4 μm in length and oriented at 40° relative to the cell circumference. ATPase activity drives the filament to extend at 85 nm/s [79]. Notably, MreB forms antiparallel double filaments on lipid membranes, inducing curvature that facilitates mechanical force propagation [6]. These membrane-bound filaments act as scaffolds to recruit effector molecules, including elongation factor Tu (EF-Tu) and phage protein p16.7, thereby positioning MreB as a central organizer of membrane-associated processes [19, 80].

#### MreB-Mediated regulation of membrane dynamics and lipid microdomain organization

MreB not only maintains cellular morphology but also modulates membrane biophysical properties, including the formation of specific membrane regions with increased fluidity (RIFs) [24]. The filament of MreB enhances the local membrane fluidity. The depletion of MreB disrupts lipid organization and causes mislocalization of membrane proteins, a functional convergence with the role of cortical actin underlying the eukaryotic cell membrane, which similarly regulates lipid raft dynamics, suggesting an evolutionarily conserved membrane-cytoskeleton crosstalk mechanism [4, 24]. Spatially, MreB is confined to the cell poles through interactions with anionic phospholipids such as phosphatidylglycerol and cardiolipin, where electrostatic repulsion governs its distribution [81]. The membrane curvature further recruits MreB, establishing a geometric feedback loop. Under stress conditions, this system activates in sequence: initially, PspA oligomers gather in cardiolipin-rich polar areas, then move laterally depending on MreB/RodZ, indicating a mechanism for sensing membrane tension [82]. Stress adaptation is tightly coupled with this network. The bacterial flotillin-like protein YqiK notably enhanced membrane fluidity, which in turn increased the mobility of MreB. This lipid-protein synergy directly impacts cytoskeletal dynamics, demonstrating how the membrane physical states modulate MreB functionality [68].

#### Metabolic integration of MreB in membrane-wall homeostasis

The MreB network tightly connects membrane dynamics with cell wall biosynthesis as a mechanochemical integrator. In *Pseudomonas mendocina* NK-01, MreB overexpression induces cellular elongation and enhances alginate oligosaccharides production by 5.86-fold compared to wild-type strains [83]; this enhancement correlates with MreB’s role in spatially organizing peptidoglycan synthases. Confocal imaging located MreB-GFP in the periplasmic space, suggesting the mechanochemical coordination between extracellular wall synthesis and intracellular metabolic flux. The evolutionary expansion of MreB’s membrane remodeling function is evidenced by its homologues directing thylakoid biogenesis in cyanobacteria and plants [82, 83]. Pathogens like *Chlamydia* employ MreB to guide polar peptidoglycan ring assembly, exemplifying its functional plasticity in replacing FtsZ-dependent division. Mechanistically, MreB interacts with the lipid II synthase MurF to enhance the polymerization efficiency, forming a feedback loop to ensure local synthesis at division sites [33, 84].

### MreB orchestrates chromosome segregation and division site positioning

MreB coordinates chromosome segregation through direct and indirect mechanisms. In *B. subtilis*, MreB anchors replisomes in mid-cell to ensure symmetric chromosome partitioning, with its depletion causing replisome mislocalization and segregation defects [85]. Although EcMreB lacks direct DNA binding, it modulates the chromosomal topological structure via topoisomerase IV regulation [86, 87]. Species-specific differences may reflect distinct chromosome organization strategies. The transcription-replication coupling via MreB-RNAP interactions facilitates the directional movement of the bacterial replication origins, while the A22-induced MreB inactivation confirmed its topological role through segregation failure [13, 38]. In *Spiroplasma*, A22 treatment reduces pathogenicity by disrupting MreB-dependent cell shape, while actin stabilizers (phalloidin) stabilize the MreB filamentous form, enhancing host cells’ invasion and cytotoxicity [88]. Notably, *Spiroplasma* MreB5 exhibits nucleotide state-dependent filament organization, with ATP hydrolysis regulating membrane binding and disassembly, offering a target for species-specific inhibitors. In cyanobacteria with multiple chromosome copies, MreB enhances the segregation fidelity through restricted diffusion [41, 89].

### Pathogen adaptation through functional modularity of the MreB network

The obligate intracellular pathogens exemplify MreB’s evolutionary plasticity. *Chlamydia* achieves FtsZ-independent division via MreB-guided polar peptidoglycan synthesis, requiring cardiolipin-driven MreB oligomerization [90–92]. The MreB-RodZ complex likely determines the segmentation plane through membrane curvature sensing [93]. Its ability to recruit FtsK demonstrates functional conservation across species, enabling MreB to maintain rod-shaped morphology while coordinating spherical cell division via the MurF-PBP2-FtsK network [11, 94]. Notably, the depletion of MreB induces cell lysis, underscoring its essential role in synchronizing peripheral peptidoglycan synthesis with septation. This property positions MreB as a promising antimicrobial target [92]. Additionally, in the pathogenic *Shigella*, MreB drives the formation of actin tails by mediating the polar localization of the virulence protein IcsA, thereby facilitating intercellular spread. When MreB polymerization is inhibited by MP265 or A22, the polar distribution of IcsA is disrupted, and bacterial motility is significantly reduced [95]. This suggests that targeting MreB can block the host-invasion mechanisms of pathogens, offering a new strategy for anti-infective therapy [95].

## MreB-Associated regulatory complexes: RodZ, MreC/D and functional partnerships

### RodZ interaction and function with MreB

RodZ interacts with MreB through the cytoplasmic and periplasmic domains, making it one of the few proteins capable of direct MreB binding [96]. This interaction enhances MreB’s circumferential rotation and polymer stability while modulating its curvature preference [97]. In *E. coli*, RodZ maintains MreB’s helical assembly pattern, ensuring the occurrence of a uniform cylindrical morphology. Deletion of RodZ causes MreB misassembly into non-helical structures, leading to severe cell shape defects, establishing RodZ as a key regulator of MreB dynamics [98].

Functionally, RodZ bridges the cytoskeleton and cell wall synthesis by coupling MreB to synthases through direct/indirect interactions, thereby coordinating synthase positioning and activity [7]. This coupling mediates MreB’s rotational motion and ensures cell wall synthesis rates match growth demands, promoting uniform cell elongation [99]. For instance, the depletion of RodZ can disrupt MreB-directed wall synthesis, uncoupling growth-division coordination [98]. RodZ further regulates MreB functionality through the geometric localization of polymers along the cell axis [99]. Its interaction with anionic lipids and MreB modulates the spatiotemporal distribution of the membrane stress sensor PspA. This multifactorial synergy enables RodZ to maintain MreB integrity and mediate mechanical stress adaptation. RodZ deletion induces MreB mislocalization, compromising membrane stress regulation and exacerbating morphological defects, ultimately positioning RodZ as the central hub linking MreB function to environmental adaptation [82].

### MreB-FtsZ functional relationship

In *E. coli*, MreB and FtsZ were found to have direct physical binding. This interaction is crucial for the contraction of the Z-ring (the constricting structure formed by FtsZ), and it also helps the cell wall synthesis enzymes transfer from the cell sidewall to the division septum to ensure the normal formation of the septum [67]. This means that the FtsZ-driven division process requires the assistance of MreB to coordinate the cell wall reconstruction, closely coupling cell elongation (the classic function of MreB) with the division event (the core role of FtsZ). For example, when the interaction between the two is disrupted, the division septum cannot be synthesized correctly, resulting in cell division failure. *Chlamydia* and other spherical bacteria lack FtsZ but retain MreB, revealing the functional plasticity of MreB under extreme conditions. Studies have found that *Chlamydia* MreB not only directly binds to key enzymes in lipid II biosynthesis such as MurF and MraY, but also can recruit other division proteins such as FtsK by forming a scaffold at the division site and replace FtsZ to become the “general commander” of division [11, 84, 94]. This functional substitution is analogous to a substitute player stepping in, whereby MreB maintains *Chlamydia* viability in the absence of FtsZ by coordinating peptidoglycan synthesis and divisome assembly.

Although the two cooperate closely, their functions also have obvious differences. FtsZ is mainly responsible for forming and contracting the fission body, while MreB usually maintains the elongation and lateral wall morphology of cells [100]. For example, in *B. subtilis*, the absence of MreB leads to cell swelling and lysis, while the defect of FtsZ directly blocks division [101]. However, certain toxins such as YeeU-YeeV (CbtA) can target both at the same time and independently inhibit cell elongation and division, indicating that they are in different branches of the same regulatory network [100]. In addition, the dynamic nature of MreB filaments is affected by FtsZ. Mutations in FtsZ alter the localization pattern of MreB, resulting in smaller circumferential bands. This suggests coordination between the two proteins through spatial positioning [92].

In some bacteria such as *Rhizobia*, abnormal interactions between MreB and FtsZ may lead to abnormal division. For example, when MreB binds to variants of FtsZ, it can impede symbiotic differentiation. This suggests that the cooperative relationship between these two proteins may need to maintain a balance in the course of evolution. Either excessive dependence or interference might result in reduced adaptability [102]. This dynamic balance is also seen in bacteria like cyanobacteria that have multiple chromosomes. MreB can assist FtsZ in completing the division by randomly distributing chromosome copies [89].

The relationship between FtsZ and MreB is akin to that of an “architect” and a “construction team”. FtsZ is like an architect who designs the blueprint for cell division, while MreB acts as the construction team, supplying building materials (peptidoglycan) and adjusting the construction direction. In the classical model, the two have a clear division of labor. However, under evolutionary pressures such as genome reduction or morphological specialization, MreB can “take on important responsibility in times of crisis” and take over part of the functions of FtsZ. This collaborative and standby relationship highlights the flexibility and robustness of bacteria in the division mechanism.

### The synergistic mechanism of the MreB/C/D complex and bacterial cell shape maintenance

MreB, MreC and MreD form the core complex that determines the bacterial cell morphology. As an actin-like protein skeleton, MreB directly binds to membrane proteins MreC and MreD, and regulates the peptidoglycan synthesis pathway through interaction with cell wall synthesis-related proteins, such as RodA, MurG and MraY [22]. MreC and MreD have clear divisions of labour during this process. MreC is responsible for the spatial arrangement of peptidoglycan synthases, such as penicillin-binding proteins and transglycosylases, in the periplasmic space, while MreD, as a transmembrane protein, is directly involved in the regulation of lateral peptidoglycan precursor synthesis and combines with precursor synthases such as MurG and MraY. These three proteins ensure the spatial coordination of cell wall synthesis through physical binding and functional complementarity [21, 22].

Not only does MreD participate in the synthesis of peptidoglycan precursors, but it also indirectly affects cell morphology by regulating the localization of MreB [21]. This interdependent localization relationship indicates that the spatial distribution of MreB requires the assistance of MreD, while the function implementation of MreD depends on the skeleton support of MreB. For example, if MreD is missing, MreB may not be able to be correctly anchored in specific regions of the cell membrane, resulting in disordered spatial organization of peptidoglycan synthesis complexes such as MurG-MraY, abnormal cell morphology [21].

**Fig. 5:**
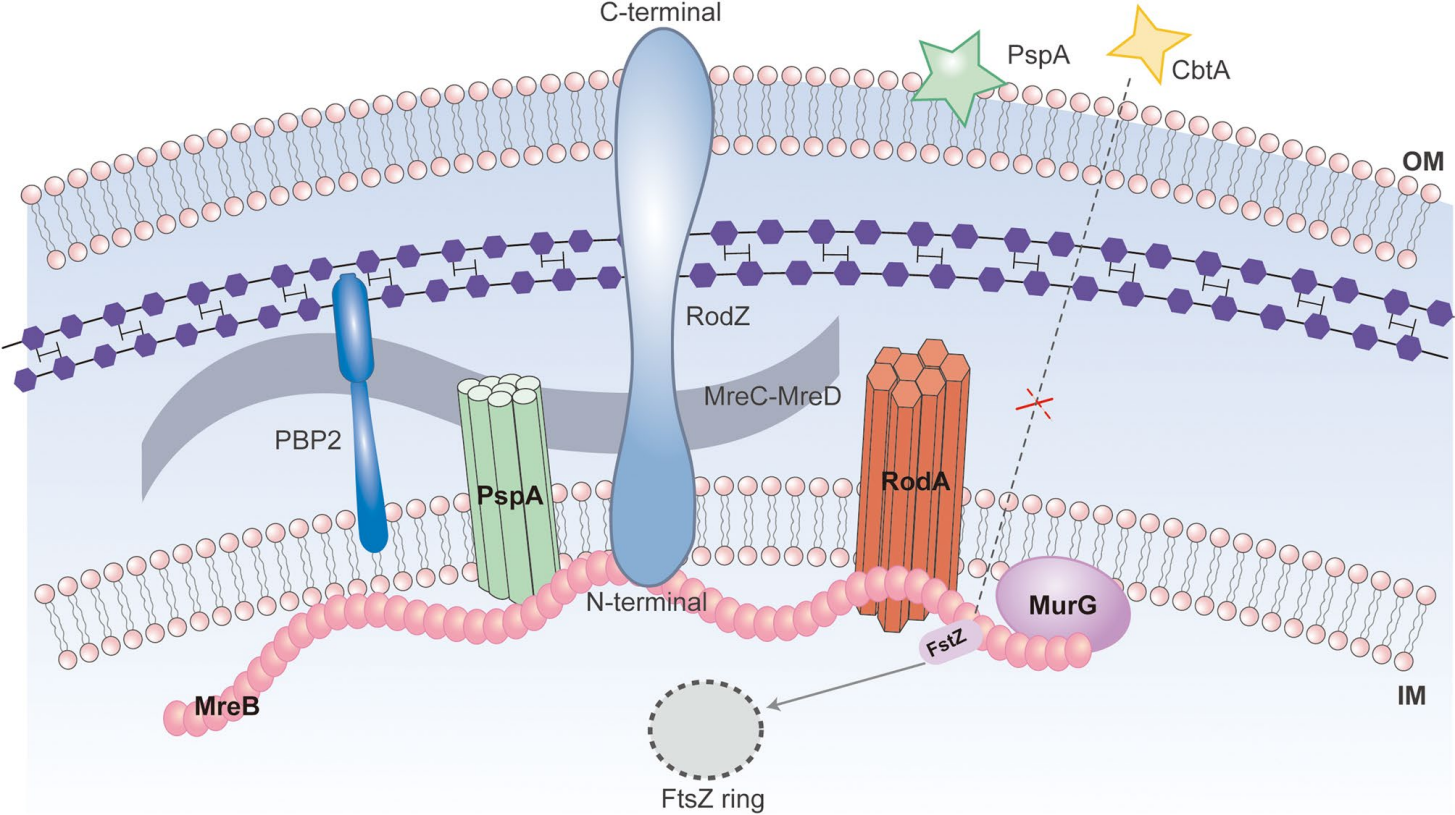
The fundamental mechanism underlying bacterial cell wall synthesis and morphological maintenance involves the interaction and function of key proteins. Green (PspA): A membrane protein regulating mechanical stress via interactions with RodZ and MreB. Red (MreB): A cytoskeletal protein coordinating cell wall synthesis, division site localization, and morphological homeostasis, collaborating with FtsZ. Dark Blue (RodZ): The main regulatory factor for MreB, connecting MreB with cell wall synthetases. Pink (FtsZ): A splitting ring protein driving division ring contraction, relying on MreB for cell wall remodeling. Orange (RodA): A peptidoglycan synthetase inserting and extending peptidoglycan chains, regulated by MreB and RodZ. Purple (MurG): A peptidoglycan precursor synthetase working with MreD. Blue (PBP2): Penicillin-binding proteins in cell wall synthesis, regulated by MreC. Yellow (CbtA): A toxin disrupting membrane pressure sensing and PspA function. Light Gray (FtsZ ring): The division ring facilitating contractile processes. Gray (MreC-MreD complex): A complex organizing peptidoglycan synthetases and regulating precursor synthesis

MreC and MreB are essential players in the geometric regulation of cell wall synthesis. MreC ensures that newly synthesized peptidoglycan chains are incorporated into the cell wall in a specific pattern by spatially orienting peptidoglycan synthases, such as cleavage transglycosylases, in the periplasmic space [22]. Concurrently, MreB directs cell wall synthesis complexes such as RodA to the designated locations on the membrane through the dynamic cytoplasmic skeletal network. The synergistic interaction between these two proteins facilitates “spatial coordination” and enhances the “efficiency” of peptidoglycan synthesis [22]. Experimental evidence indicates that depletion of MreC or MreB can result in cells adopting a rounded morphology due to their inability to maintain structural rigidity, thereby underscoring the crucial role of these two proteins in maintaining the geometry of cells [103]. While Mbl, a homolog of MreB, is involved in chromosomal segregation, MreC, together with MreD and MreBH (found in *B. subtilis*), primarily concentrates on preserving cellular shape [103]. The functional specificity associated with complexes containing MreB is illustrated by distinct morphological changes observed upon their depletion; for instance, the loss of both MreC and MreD leads to a transition from rod-shaped to globular forms, while the absence of MreBH results in a *Vibrio*-like curved phenotype [103]. Through mechanisms independent of chromosome segregation processes, the cooperative action between the MreC/MreD/MreB complex regulates three fundamental aspects: cell wall biogenesis, division site localization, and maintenance of shape homeostasis across spatiotemporal dimensions. This diverse function includes roles such as shape - determining coordinators (defining cell geometry), synthetic frameworks (directing peptidoglycan biosynthesis), and molecular integrators (building interaction networks), thereby ensuring bacterial survival adaptation and their responsiveness to environmental stimuli (Fig. 5).

## Environmental modulation of MreB: connecting polymerization dynamics to pathogenic adaptation

### Temperature and ionic cues modulate MreB polymerization dynamics

MreB’s polymerization behavior is strongly influenced by environmental signals, especially temperature and ion concentration. Studies indicate that the ultrastructure and polymerization of MreB filaments are heavily dependent on these factors [104]. For example, TmMreB polymerization rate increases significantly with rising temperature, its critical concentration decreases, and it assembles over a wide temperature range. This is distinct from the conclusion of the traditional view that high temperature inhibits polymerization [28]. However, temperature does not act in isolation: monovalent salts such as K⁺ and Na⁺ can inhibit polymerization, while divalent cations such as Mg²⁺ and Ca²⁺ promote nucleation and elongation through a staged mechanism [28, 37]. Interestingly, although BsMreB can be assembled without nucleotides, its polymerization is still inhibited by pH value and monovalent salts, suggesting that different bacterial strains may have evolved differentiated environmental response strategies [37].

Anionic phospholipids, such as phosphatidylglycerol and cardiolipin, act as ‘gatekeepers’ of spatial localization, indirectly controlling cell morphology by regulating the subcellular localization of MreB. In *E. coli*, these lipids are enriched in the cell poles and preferentially bind to monomeric MreB, thereby excluding the polymerized MreB outside the polar region and maintaining the polar growth of rod-shaped cells [81, 105]. If anionic phospholipids are absent, MreB will be abnormally localizes at the cell poles, leading to the formation of Y-shaped cells during cell division [105]. Further research has found that anionic phospholipids may affect the movement speed of MreB-related complexes, such as the Rod complex by changing membrane fluidity. Moreover, changes in membrane fluidity, such as high temperature or fatty acid synthesis defect can directly interfere with the function of these complexes, leading to abnormal cell morphology [106]. This lipid-protein interaction highlights the close coupling between the physical properties of the membrane and the dynamics of the cytoskeleton.

### The synergistic effect between nucleotides and cations

The nucleotide-binding state and cation concentration jointly shape the assembly mode of MreB. For instance, TmMreB requires binding to purine nucleotides (ATP/GTP) to polymerize, for polymerization, yet following assembly, it rapidly hydrolyzes ATP to ADP. Consequently, most MreB filaments within the cell exist in an ADP-bound state [28]. This dynamic hydrolysis may endow the filaments with reversible structural changes. When ADP or GDP is present, MreB tends to form parallel linear protofilaments, while ATP may promote the formation of sheet or bundle structures by regulating electrostatic interactions [16, 107]. Additionally, cation concentrations, particularly those of K⁺ and Na⁺, influence the polymerization rate and determine the morphology of the filament, specifically, bundle structures form when ion concentrations are low, while sheet structures form at high concentrations [16]. This ion dependence has even been developed into a tool for measuring the concentration of cations within prokaryotic cells [108].

### Niche-specific adaptations of MreB signaling networks

**Fig. 6:**
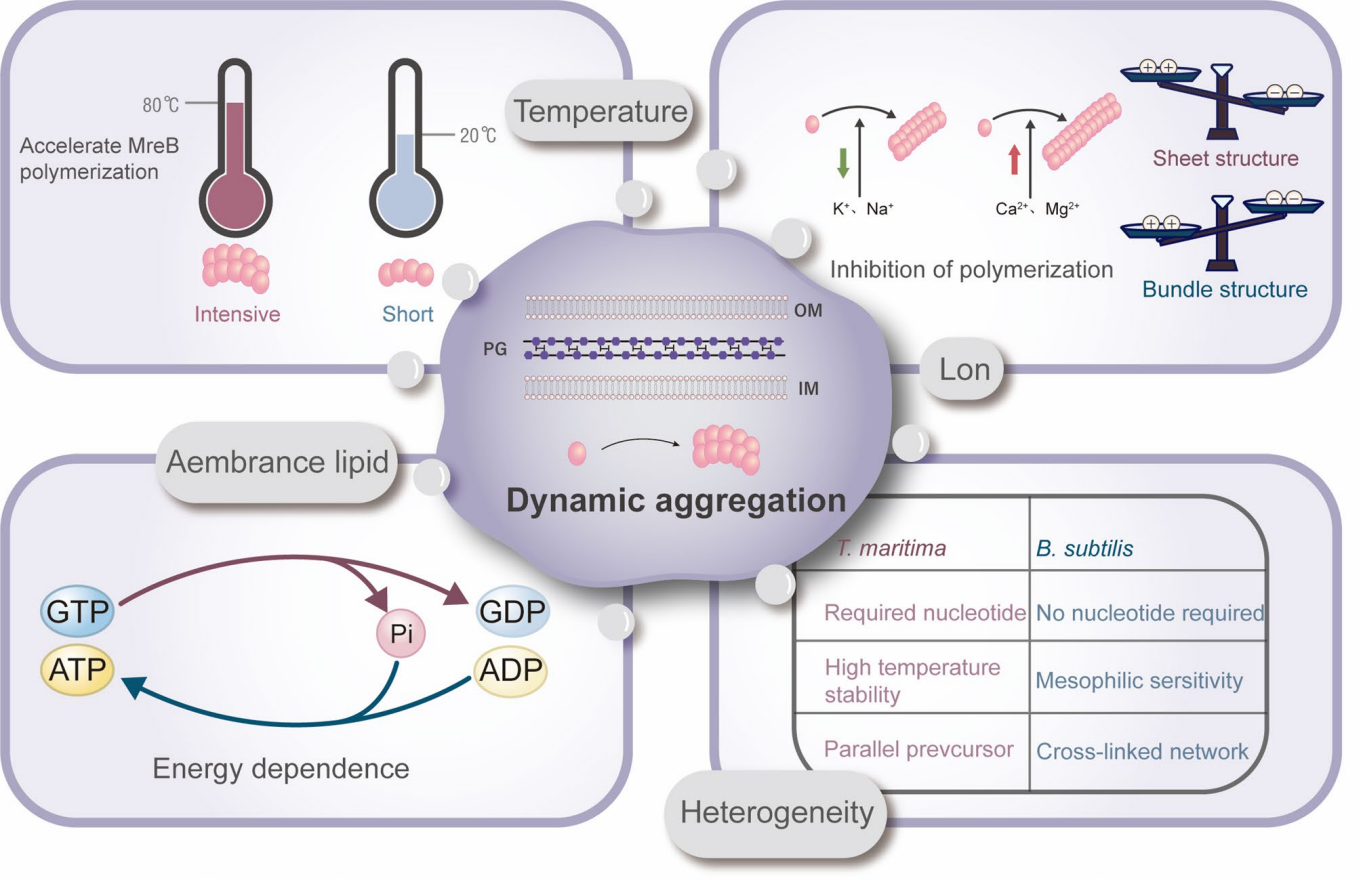
Dynamic regulation network of MreB polymerization and species heterogeneity. MreB polymerization is regulated through temperature-dependent feedback mechanisms, such as the reduction of critical concentration at high temperatures by *T. maritima* to facilitate stable assembly. It is also influenced by ionic gradient modulation, where monovalent ions inhibit nucleation while divalent ions promote elongation, with ion concentrations determining the microstructure morphology. Additionally, MreB polymerization involves coupling with nucleotide metabolism: ATP and GTP drive the initiation process, ADP helps maintain equilibrium, and the type of nucleotide dictates structural transitions. Different species exhibit heterogeneity: *Bacillus* depends on high divalent ion concentrations and pH sensitivity, *S. pneumoniae* forms pH-Mg²⁺-dependent lateral interactions via its positively charged C-terminal domain, and *T. maritima* relies on nucleotide cycling for low critical concentration adaptation across temperatures. These findings highlight the evolutionary plasticity and environmentally adaptive molecular design in prokaryotic morphogenesis networks

MreB exhibits marked functional diversity in environmental signals across different bacterial species, reflecting its complex regulatory roles. For instance, when polymerizing independently of nucleotides, BsMreB requires micromolar concentrations of divalent cations, with a critical concentration of ~ 900 nM [37]. In contrast, TmMreB strictly depends on nucleotides for polymerization, operating at a significantly lower critical concentration [28]. *Streptococcus pneumoniae* (*S. pneumoniae*) MreB leverages a positively charged C-terminal domain to mediate lateral interactions, and assembly requires specific pH values and Mg²⁺ conditions [16]. In *S. eriocheiris*, the assembly of MreB5 depends on pH value and ions: under acidic conditions, it inhibits lamellar formation but promotes Mg²⁺-dependent aggregation, while its disordered C-terminal region enhances lateral interactions [16]. Such environmental sensitivity aligns with MreB’s role in adapting to host niches, as evidenced by the reduced pathogenicity of *B. subtilis* following treatment with actin stabilizers or the MreB-specific inhibitor A22 [88]. These cross-species variations likely represent evolutionary adaptations to ecological niches, underscoring the limitations of relying on a single in vitro model to replicate native in vivo dynamics [109] (Fig. 6).

## Pharmacological targeting of MreB: from inhibitors to therapeutic strategies

A22 was the first widely studied small molecule inhibitor of MreB. Its mechanism of action revealed the dynamic regulation of environmental signals on the bacterial cytoskeleton. This S-benzyl isothiourea compound does not directly kill bacteria. Instead, it competitively inhibits the binding of ATP to MreB (similar to the ADP-bound state), forcing the rapid depolymerization of MreB filaments in vivo [38]. Interestingly, this “signal interference” does not completely block the synthesis of the cell wall, but instead leads to the transformation of *E. coli* from rod-shaped to spherical, suggesting that environmental pressure may reshape the bacterial morphology through conformational changes of MreB [110]. The particularity of A22 lies in providing a non-lethal intervention model and opening up the path for the developing of new anti-infective drugs targeting the cytoskeleton [38].

Compound A found in *Pseudomonas aeruginosa* (*P. aeruginosa*) further verified the universality of this regulatory strategy. Although it is highly similar in structure to A22, the resistance screening of compound A showed that its activity is unaffected by efflux pumps, which is particularly important for clinical drug resistance problems. The morphological transition from rod-shaped to spherical, induced by environmental cues, may diminish the bacteria’s capacity to invade host cells and decrease endotoxin release. This implies a profound connection between the mechanical signals conveyed by MreB and bacterial pathogenicity. More interestingly, analogues such as 9 and 10, even exceed the ATPase inhibitory potency of the classic inhibitor CBR-4830 (with an IC50 as low as 6 ± 2 µM) for EcMreB, indicating that chemical modification can finely regulate the action strength of such environmental signaling molecules [111, 112].

The regulation of MreB by environmental signals is not limited to exogenous compounds; bacteria have also evolved endogenous regulatory systems. The newly discovered membrane-binding toxin CptA (YgfX) directly blocks the polymerization of MreB and FtsZ through its cytoplasmic domain [113]. This “suicidal” regulation leads to cell swelling and a lemon-shaped appearance. As the first membrane-associated TA system toxin, the transmembrane segment of CptA may sense physical signals such as membrane tension and then transmit them to the cytoskeletal network through MreB [113]. This cross-regulatory network of internal and external signals suggests that the interfering with the bacteria’s stress response mechanisms should be considered in the design of drugs targeting MreB.

Current research reveals that both exogenous compounds (A22/Compound A) and endogenous toxins (CptA) achieve regulation by changing the nucleotide-binding state of MreB. This “signal hijacking” strategy shows potential in combined therapy. The synergistic effect of A22 with clinical antibiotics, or the derivatives obtained through structural optimization with anti-efflux pump properties all provide new ideas for addressing the crisis of drug resistance [112, 114]. The molecular mechanism of A22 involves its competitive binding to a site adjacent to MreB’s nucleotide-binding pocket, effectively inhibiting ATP hydrolysis and destabilizing the antiparallel protofilament dimer structure [38, 39]. This precise targeting of a functionally critical site provides a foundation for rational drug design aimed at disrupting MreB dynamics. The discovery of MreB inhibitors often uses strategies similar to those for FtsZ-targeting compounds. These include phenotypic screening for morphological changes like rod-to-sphere transition, biochemical assays for ATPase inhibition or polymerization disruption, and structure-based virtual screening against the conserved nucleotide-binding pocket to identify novel scaffolds.

**Fig. 7:**
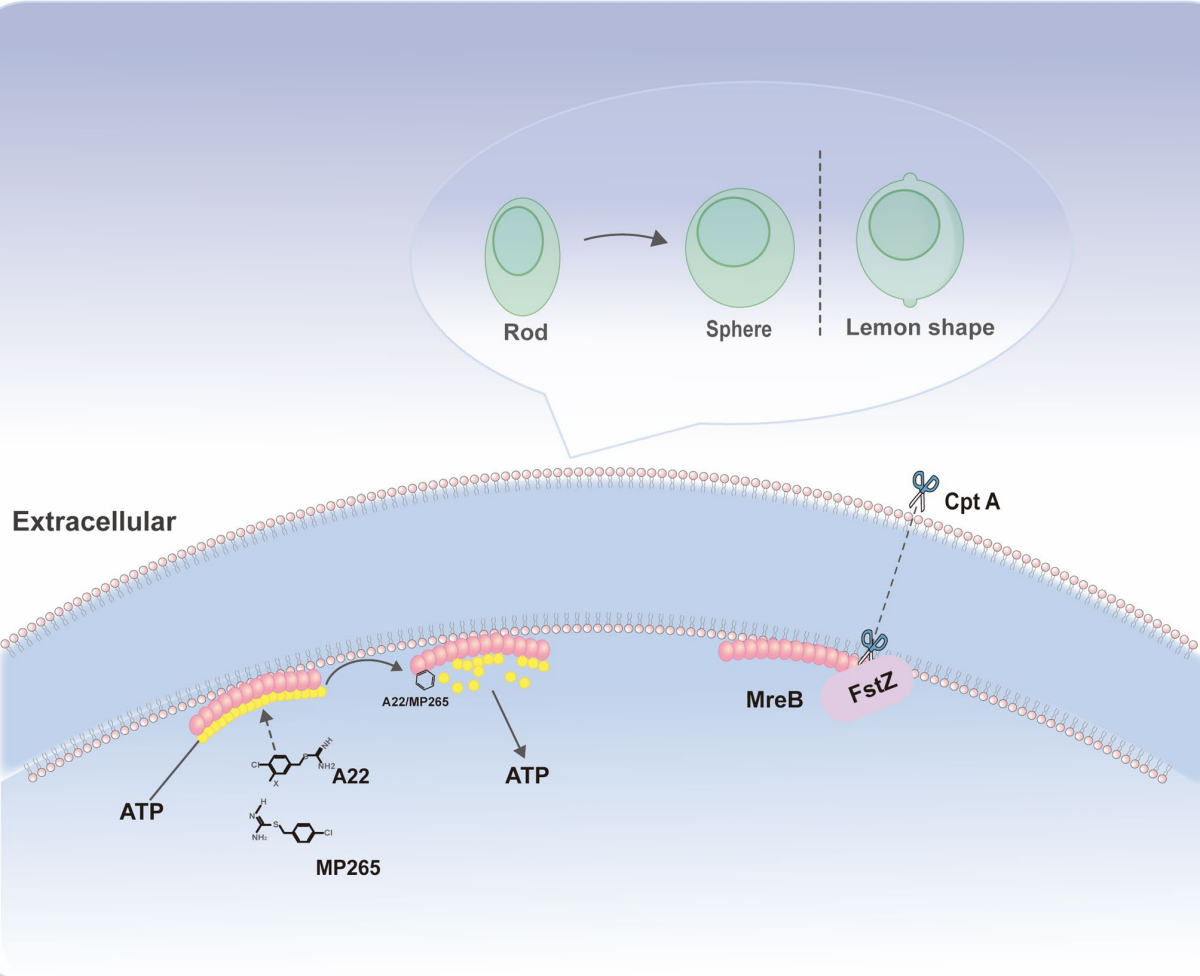
Pharmacological Targeting of MreB and Resulting Cellular Morphological Changes. A22 and Mp265 competitively inhibit the binding of ATP to MreB, causing rapid depolymerization of MreB filaments in vivo and leading to a morphological shift of *E. coli* from rod-shaped to spherical. In contrast, CptA (YgfX) directly blocks the polymerization of MreB and FtsZ, resulting in cell swelling and a lemon-shaped appearance in *E. coli*

Future research requires in-depth analysis of how environmental signals couple cell morphology and metabolic pathways through changes in the ATPase activity of MreB, which will promote the development of more precise “signal-skeleton” regulatory drugs [115] (Fig. 7).

The diversity of MreB-targeting compounds underscores its potential as a therapeutic target. We summarize the key pharmacological agents, their specific targets, mechanisms of action, and resultant antibacterial activities (Table 1). These inhibitors exploit distinct vulnerabilities, competitive ATP binding inhibition (A22, Compound A), ATP hydrolysis blockade and filament destabilization (MP265, CBR-4830), dual inhibition of MreB and FtsZ polymerization (CbtA/CptA), or filament stabilization (Phalloidin). This spectrum of mechanisms leads to characteristic morphological defects such as rod-to-sphere transition, lemon-shaped deformation and attenuation of virulence, highlighting the critical role of a functional MreB cytoskeleton in bacterial survival and pathogenicity.

**Table 1:** Antimicrobial molecules targeting MreB and their mechanisms of action

Molecule Name	Target Specificity	Mechanism of Action	Antibacterial Activity
MP265 (A22 analog)	Directly targets MreB	Binds adjacent to MreB’s nucleotide-binding site, inhibits ATP hydrolysis, and destabilizes antiparallel protofilament dimers	Blocks polar localization of virulence protein IcsA in *Shigella*, significantly reducing motility and host invasion
A22 (S-benzyl isothiourea)	Directly targets MreB	Competitively inhibits ATP binding to MreB, mimics ADP-bound state, and induces filament depolymerization	Induces rod-to-sphere transition in *E. coli* and *Shigella*, attenuating virulence
Compound A (A22 analog)	Directly targets MreB	ATP-competitive inhibition mechanism similar to A22; evades efflux pump-mediated resistance	Effective against *P. aeruginosa*; induces morphological defects
CbtA/CptA	Dually targets MreB & FtsZ	Membrane-bound toxin whose cytoplasmic domain directly inhibits polymerization of both MreB and FtsZ	Induces cell swelling and lemon-shaped deformation, leading to lysis
CBR-4830	Directly targets MreB	ATPase inhibitor (IC₅₀ = 6 ± 2 µM); disrupts dynamic assembly	Effective against Gram-negative bacteria
Phalloidin	Stabilizes MreB filaments	Binds and stabilizes MreB polymers (particularly in *Spiroplasma*)	Enhances *Spiroplasma* pathogenicity; low selectivity for bacterial cells

## Conclusion

MreB is a key part of bacterial cellular organization, joining cytoskeletal dynamics with cell wall synthesis, membrane remodeling, and chromosome segregation. Its structural homology with actin belies unique functional adaptations: anti-parallel protofilament assembly, nucleotide-state-dependent membrane interactions, and species-specific roles, such as guiding lipid II synthases in *Chlamydia* or driving helical motility in *Spiroplasma*. Environmental plasticity enables MreB to rapidly adapt to ionic gradients, temperature shifts, and membrane composition changes, positioning it as a central hub for stress survival. Pharmacologically, small-molecule inhibitors like A22 target the nucleotide-binding dynamics of MreB, inducing morphological aberrations and attenuating virulence in pathogens like *Shigella flexneri*. Yet, its evolutionary versatility is most striking: from replacing FtsZ for division to enabling synthetic motility systems, MreB demonstrates how conserved modules can be repurposed across biological contexts. We integrate this functional diversity and emphasized how MreB compensates for the absence of certain cellular mechanisms in evolutionarily distinct bacteria (Table 2).

**Fig. 8:**
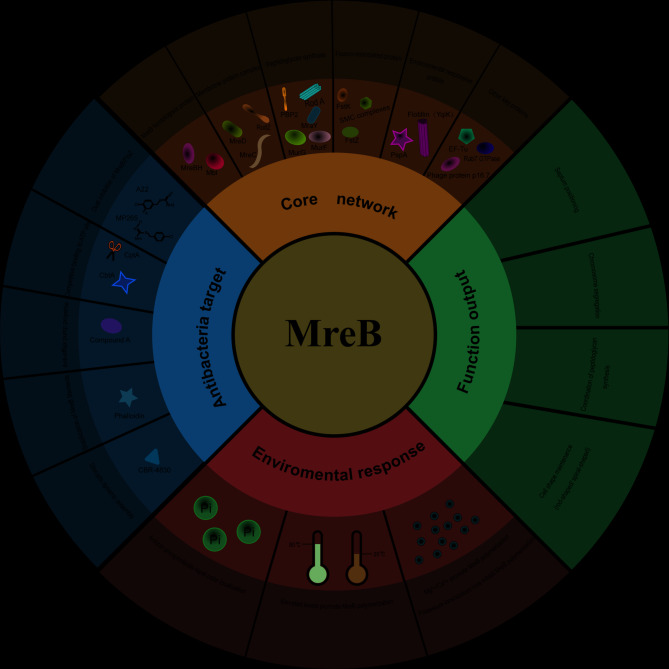
MreB interaction network and functional outcomes. MreB’s role in core network formation, antibacterial targeting, environmental responses, and function outputs, detailing specific interactions and outcomes

To synthesize these multifaceted roles and interactions, we propose a conceptual framework of the MreB system, encompassing its core network, functional outputs, environmental responsiveness, and pharmacological targeting (Fig. 8). MreB serves as the central scaffold of a complex network involving key partners including RodZ, MreC, and FtsZ. This network coordinates fundamental cellular processes, including morphogenesis, peptidoglycan synthesis, chromosome segregation, and division site positioning. Critically, MreB’s function is modulated by environmental cues such as ions, temperature, lipids and can be disrupted by specific inhibitors, such as A22, MP265, Compound A, CptA, CBR-4830 and Phalloidin, leading to loss of shape control and virulence.

This review consolidates two decades of research, highlighting MreB’s dual identity as a structural scaffold and a regulatory nexus. Furter investigations, resolving its mechanobiological principles and ecological interactions will be essential to unlock its full therapeutic and biotechnological potential.

**Table 2:** Functional diversification of MreB paralogs in bacteria

Category	Typical Species	Protein	Classical Function	Acquired Function	Driving Deficiency		Evolutionary Driver
Rod-shaped bacteria	*E. coli*, *B. subtilis*	MreB, Mbl, MreBH	Cell shape maintenance and peptidoglycan synthesis coordination	Chromosome segregation via RNA polymerase (RNAP) coupling and replisome anchoring		None (ancestral function)	Optimization of cytoskeletal multitasking
Wall-less bacteria	*Spiroplasma*	MreB1–MreB7 (such as MreB5)		Helical motility, kink propagation, and membrane deformation		Absence of cell wall	Loss of rigid wall enables mechanical force-driven motility
FtsZ-deficient pathogens	*Chlamydia*	MreB	Peptidoglycan ring assembly and lipid II synthase recruitment	FtsZ-independent division and divisome scaffolding		Absence of FtsZ	Genome reduction in obligate intracellular pathogens
Morphologically plastic bacteria	*S. coelicolor*	MreB	Vegetative hyphae shape	Spore-specific wall remodeling		Developmental reprogramming	Lifecycle adaptation (hyphae to spores)
Morphologically plastic bacteria	*C. crescentus*	MreB	Cell elongation	Stalk synthesis		Localized divisome redistribution	Niche adaptation (polar growth)
Pathogen virulence	*S. flexneri*	MreB	Cell shape maintenance	Actin tail formation (via IcsA)		Host invasion mechanism	Pathoadaptation for intercellular spread

## Future perspectives

Bacterial actin homolog MreB exemplifies the evolutionary adaptability of conserved molecular scaffolds. While its roles in morphogenesis, peptidoglycan synthesis and chromosome segregation are well established, critical gaps remain in understanding its mechanochemical regulation [11, 116]. For instance, how the MreB-RodZ complex converts membrane curvature into localized peptidoglycan synthesis remains unresolved [7, 97]. Advanced techniques, such as molecular dynamics simulations combined with optical tweezers, can map force-dependent conformational changes, while cryo-electron tomography may resolve native-state filament architectures across different species [6, 109]. Addressing these challenges will require integrating advanced imaging techniques such as cryo-electron tomography, with synthetic biology approaches. This integration will enable a detailed dissection of MreB’s mechanochemical signaling networks [109]. The structural differences of MreB homologs, like nucleotide-dependent polymerization in *T. maritima* and nucleotide-independence in *B. subtilis*, show the need to conduct comparative studies to develop species-specific antimicrobials [28, 37]. Recent work on *Chlamydia* MreB, which compensates for FtsZ absence by coordinating lipid II synthase activity, underscores its potential as a therapeutic target [11, 91]. However, challenges persist: MreB-depleted *E. coli* develops β-lactam tolerance via efflux pumps, necessitating combinatorial therapies [101, 115]. Emerging tools like optogenetics and synthetic biology approaches could further harness MreB’s modularity for biotechnological applications, such as programmable morphogenesis or enhanced metabolite production [66, 75, 83]. Future efforts must also address ecological and evolutionary dimensions. Laboratory evolution under antibiotic stress and metagenomic analyses of environmental niches may reveal conserved stress responsive motifs [78]. These insights will refine phylogenetic modeling and resistance prediction. Furthermore, MreB potentially assumes critical functions in bacteria without a cell wall or lacking most proteins from the divisome complex. Future studies could be directed towards investigating this area. This comprehensive overview establishes the framework for future research on the structure and construction of microorganisms.

## Data Availability

No datasets were generated or analysed during the current study.
